# Predictive Validity of the Brøset Violence Checklist in a Secured Institution for Offenders With Intellectual Disabilities

**DOI:** 10.1111/jir.13233

**Published:** 2025-03-20

**Authors:** Jacob Hvidhjelm, Søren Holst

**Affiliations:** ^1^ Psychiatric Research Unit Mental Health Services Region Zealand Denmark; ^2^ Department of Sociology and Social Work Aalborg University Aalborg Denmark; ^3^ The Secured Social Residential Unit Kofoedsminde Region Zealand Denmark

**Keywords:** Brøset violence checklist, intellectual disabilities, offender management and violence prediction, risk assessment, secured institutions

## Abstract

**Background:**

The integration of systematic risk assessments into care settings for offenders with intellectual disabilities (IDs) is a growing priority, yet evidence on the validity of existing tools in this population remains limited. The Brøset Violence Checklist (BVC) is widely used to assess short‐term violence risk, but its predictive accuracy in individuals with IDs remains uncertain. This study investigates the predictive validity of the BVC in a specialized institutional context for offenders with IDs, focusing on its performance across different shifts and demographic subgroups.

**Method:**

A retrospective observational design was used to analyse 153 262 BVC assessments and 1325 documented severe violent incidents over 2.5 years in a secured Danish institution for offenders with IDs. Sensitivity, specificity, positive predictive value (PPV) and negative predictive value (NPV) were calculated across different BVC thresholds. Generalized linear mixed models (GLMMs) were applied to assess the influence of sex and time of day on predictive performance.

**Results:**

The BVC demonstrated high specificity and NPV across all shifts, confirming its utility in identifying low‐risk scenarios. However, sensitivity and PPV were limited, particularly during night shifts and at higher score thresholds. Predictive accuracy was highest during evening shifts, aligning with periods of increased staff–resident interactions. GLMM analyses indicated that the relationship between BVC scores and violence risk was moderated by sex and time of day, with female residents and low‐activity periods presenting unique challenges to prediction.

**Conclusions:**

The findings underscore the need for population‐specific adaptations to the BVC, particularly to address contextual and demographic factors influencing aggression in ID populations. Recommendations include supplemental assessment strategies for low‐activity shifts, sex‐specific behavioural indicators and the development of tailored tools for ID care settings. This study advances the understanding of violence risk dynamics in ID populations and informs interventions to enhance safety for residents and staff.

## 1 Introduction

In Denmark, as is the case in many Western countries, risk assessments have been increasingly integrated into psychiatric care in recent decades (Hvidhjelm et al. 2023). However, risk assessments in institutions serving offenders with intellectual disabilities (IDs) remain a relatively unexplored area. In most countries, offenders with IDs are either incarcerated or treated in psychiatric facilities (Tromans et al. 2023). In contrast, Denmark operates under a distinct legal framework where individuals with an ID are not considered to be criminally responsible and are therefore sentenced to care and treatment rather than punishment (Justitsministeriet 2024). These individuals are placed in residential units that are staffed by social pedagogical personnel under the Danish Consolidation Act on Social Services (Social‐ og Boligministeriet 2024).

The Kofoedsminde (KFM) institution, located in southeastern Denmark, is the sole secured residential facility for offenders with IDs who have committed severe offences, such as murder, rape or arson. Unlike prisons or hospitals, KFM operates under a social pedagogical care model that emphasizes integration, education and ethical relationships between staff and residents. This reflects Denmark's historical shift in the 1970s and 1980s, whereby the responsibility for individuals with IDs transitioned from healthcare to social care, with chief physicians being replaced by social work–trained principals (Madsen 2005).

### 1.1 Workplace Violence and Demand for Risk Assessment

Concerns about workplace violence have driven the adoption of systematic risk assessments in social care institutions. The Danish Working Environment Authority (Arbejdstilsynet) highlights the importance of violence prevention in workplaces, particularly in care settings where staff may be at a heightened risk because of the challenging behaviours of the residents (Arbejdstilsynet, n.d.). Between 2012 and 2018, five homicides targeting social pedagogical staff occurred in psychiatric care units. Although no such incidents were reported in ID care settings, these events prompted an increased demand for violence prevention measures, including risk assessment tools, to ensure staff safety across various institutions (Beskæftigelsesministeriet 2020; Holst 2018).

The Brøset Violence Checklist (BVC), originally developed for psychiatric care (Almvik et al. 2000), has become the most commonly used tool in Danish social institutions, including KFM. The BVC evaluates the imminent risk for violence based on six behavioural indicators and has been validated extensively in psychiatric populations (e.g., Abderhalden et al. 2004; Bjorkdahl et al. 2006; Hvidhjelm et al. 2014). However, its validity in populations with IDs remains largely untested. Although studies in psychiatric settings report high predictive accuracy, including a pooled AUC of 0.826 in a recent meta‐analysis (Hvidhjelm et al. 2023), comparable data for ID populations are lacking, raising concerns about their applicability.

At KFM, the BVC has been used routinely since 2009, alongside the Staff Observation Aggression Scale–Revised (SOAS‐R) (Nijman et al. 1999). In 2014, KFM expanded its risk assessment toolbox to include the HCR‐20 (Historical, Clinical, Risk Management‐20), a structured tool that has been designed to evaluate long‐term violence risk (Cheng et al. 2019; Webster et al. 1997).

### 1.2 Knowledge Gap

Despite its widespread use in Danish ID institutions, the BVC has not been validated for this specific population, raising concerns about its reliability and utility. The behavioural indicators assessed by the BVC may manifest differently in individuals with IDs, potentially affecting its sensitivity, specificity and predictive accuracy. Empirical validation is essential to ensure that risk assessment tools are evidence‐based and tailored to the needs of individuals with IDs.

Temporal and environmental factors, such as time of day and interaction levels, significantly influence violence risk assessments. Tools like the BVC perform better during high‐interaction periods (e.g., evening shifts) compared with low‐activity periods (e.g., night shifts) (Lockertsen et al. 2020; van de Sande et al. 2011). Additionally, sex differences in aggression manifestation challenge the BVC's generalizability in mixed‐gender populations, as women often exhibit aggression through verbal or self‐directed behaviours rather than physical threats (Dickens et al. 2020; Eriksen et al. 2018). Understanding these contextual and demographic influences is crucial for adapting the BVC to specialized settings, such as institutions for individuals with IDs.

### 1.3 Aims

This study investigates the predictive validity of the BVC in a specialized institution for offenders with intellectual disabilities, focusing on its sensitivity and specificity in predicting violent incidents within 24 h. It also examines how contextual factors, such as time of day, moderate its predictive performance.

## 2 Methods

### 2.1 Study Design

The study followed a retrospective observational design, utilizing data routinely collected as part of care at a single Danish institution.

### 2.2 Setting and Population

KFM operates under the Danish Consolidation Act on Social Services and follows a social pedagogical care model emphasizing education and integration. Residents are housed under secure custody because of severe offences, such as murder, arson and rape but are exempt from criminal punishment under the Danish Penal Code because of their intellectual disabilities (Justitsministeriet 2024, §16, stk. 2). The institution is staffed by social educators, psychologists and care professionals who are trained to ensure safety and support rehabilitation while managing challenging behaviours.

The study population consisted of all residents living at KFM during a period of 2.5 years (April 2017–September 2019). A total of 102 residents were included, of whom 92 (90%) were male and 10 (10%) were female. The median age was 35 years (mean: 35.8 years; SD: ± 11.47). Most residents have mild intellectual disabilities (IQ: 50–69), with 74.7% having been diagnosed with additional psychiatric disorders, including autism spectrum disorder (F84), attention‐deficit/hyperactivity disorder (ADHD; F90.0) and behavioural or attachment disorders (World Health Organization 2004). They reside in this facility because of committing severe crimes like violence (73.5%), sexual offences (21.5%) and arson (37.4%). Most have troubled upbringings, with 80% experiencing abuse or neglect. Stays average 6.7 years, with some exceeding 20 years, reflecting ongoing risk assessments.

### 2.3 Behavioural Data Collection

Data on residents' behaviour were collected using the BVC (Almvik and Woods 1998; Linaker and Busch‐Iversen 1995) and the SOAS‐R (Nijman et al. 1999).

The BVC is a structured risk assessment tool that is designed to predict imminent violent behaviour. It evaluates six behavioural indicators—confusion, irritability, boisterousness, physical threats, verbal threats and attacks on objects. The six indicators are scored according to whether they are absent (score = 0) or present (score = 1); then, they are subsequently summed. Scores range from 0 to 6, with 0 indicating low risk, 1–2 indicating moderate risk and 3 or higher indicating high risk, warranting immediate intervention (Woods and Almvik 2002). At KFM, the BVC was integrated into the standard daily routine and administered three times per day at the end of each shift: during the day shift (07:00–15:00), the evening shift (15:00–23:00) and the night shift (23:00–07:00), each lasting 8 h. All staff members were trained to implement preventive interventions in accordance with both local and original guidelines (Almvik and Woods 1998).

Violent incidents were documented using the SOAS‐R, a validated tool for recording aggression towards people or objects. The study adopted Morrison's (1990) definition of aggression, characterizing it as behaviours involving ‘any verbal, nonverbal or physical behaviour that was threatening (to self, others, or property), or physical behaviour that actually did harm (to self, others, or property)’. The SOAS‐R assigns severity scores ranging from 0 to 22 (Nijman et al. 1999, 2002), with incidents scoring 9 or more being classified as severe violence (Abderhalden et al. 2004; Almvik et al. 2000). The SOAS‐R is completed as close to the observation of the aggressive incident as possible to ensure accurate documentation of the event and its context. All staff members received formal training on the use and documentation of the BVC and SOAS‐R, including when, how and why these tools should be applied, ensuring consistency in scoring and registration across all shifts.

To maintain data independence, BVC scores and SOAS‐R incidents occurring within 24 h of a previous violent episode or a BVC score ≥ 1 were excluded. This ensured that repeated events were not double‐counted as separate incidents (Lockertsen et al. 2021). Each violent event was linked to the most recent BVC assessment prior to its occurrence, ensuring that predictions were based on relevant and independent observations (Hvidhjelm et al. 2014). If a BVC score ≥ 1 was recorded, subsequent BVC assessments within 24 h were excluded to avoid overlapping predictions. Similarly, if a violent incident occurred, additional SOAS‐R episodes within the next 24 h were excluded as part of the same escalation. Data collection resumed after the 24‐h period.

### 2.4 Statistical Analysis

Statistical analyses included descriptive statistics, receiver operating characteristic (ROC) analysis to evaluate sensitivity and specificity at different thresholds and generalized linear mixed models (GLMMs) to assess interactions between BVC scores, sex and shifts (day, evening and night). The predictive validity of the BVC was assessed using a combination of descriptive statistics, ROC analysis and GLMMs. All analyses were conducted using R (version 4.4.2).

### 2.5 Descriptive Statistics

The distribution of BVC scores was examined to provide an overview of the dataset. The proportion of assessments with scores of 1 or more, 3 or more and other thresholds was calculated, along with the frequency of individual behavioural indicators, such as irritability, confusion and boisterousness. Violent incidents, which were documented using the SOAS‐R, were categorized by severity (e.g., SOAS‐R ≥ 9 indicating severe violence) and stratified according to time of day (day, evening or night) to identify potential temporal patterns. These descriptive analyses formed the basis for subsequent predictive modelling and ROC analysis.

### 2.6 ROC Analysis

The ROC analysis was performed to evaluate how well the BVC distinguished between violent and nonviolent outcomes. For each threshold of the BVC (e.g., ≥ 1, ≥ 2 and ≥ 3), the sensitivity, specificity, positive predictive value (PPV) and negative predictive value (NPV) were calculated. These metrics provided a detailed assessment of the tool's ability to predict violent behaviour:

- **Sensitivity** measures the proportion of violent incidents correctly identified.
- **Specificity** reflects the proportion of nonviolent incidents correctly classified.
- **PPV and NPV** assess the practical utility of the tool for predicting violence and ruling out nonviolence, respectively.

The ROC curve was constructed by plotting sensitivity against (1‐specificity) across all thresholds. The area under the curve (AUC) was computed as a single summary measure of the BVC's predictive accuracy, with values closer to 1 indicating better discriminative performance. Additionally, the analysis was stratified according to shift (day, evening or night) to explore temporal variations in the tool's predictive validity.

### 2.7 GLMMs

A GLMM approach was used to examine the relationship between BVC scores and violent behaviour while accounting for repeated measures and individual variability. Violent behaviour, recorded as a binary outcome (yes = 1; no = 0), served as the dependent variable. The main predictor was the BVC score, treated as a continuous variable. Fixed effects included sex, shift (day, evening or night) and their interactions with BVC scores; these were used to assess how these factors influenced the predictive strength of the tool.

Random effects were included to account for individual differences in baseline violence risk. Specifically, a random intercept was modelled for each resident to capture variability that could not be explained by the fixed effects. This approach allowed the model to adjust for the repeated nature of the data, as residents often had multiple assessments over time.

The GLMM was fitted using the ‘lme4’ package in R, employing a binomial distribution and logit link function. Maximum likelihood estimation (via Laplace approximation) was used to estimate parameters. Model performance was evaluated using the Akaike information criterion (AIC), with lower values indicating a better model fit. Scaled residuals were examined to ensure the model adequately captured the variability in the data.

### 2.8 Predicted Probabilities

To provide actionable insights, the predicted probabilities of violence were calculated from the GLMM for different BVC scores. These probabilities were adjusted for sex (male or female) and shift (day, evening or night). For example, the model estimated the likelihood of violence for a male resident scoring 3 on the BVC during the evening shift compared with a female resident scoring the same during the day shift. These probabilities helped contextualize the practical implications of BVC scores for violence risk assessment.

### 2.9 Software

All analyses were conducted in R (version 4.4.2). Key packages included lme4 for GLMM modelling and pROC for ROC analysis. Visualizations, such as ROC curves and predicted probability plots, were created using ggplot2.

### 2.10 Ethical Approvals

This study was conducted in accordance with the principles of the Declaration of Helsinki (World Medical Association 2024). As the study relied on anonymized data, which were collected as part of routine care, formal ethical approval was not required. However, institutional approval was obtained from KFM's management. To ensure anonymity, no personally identifiable information was included, and individual residents could not be traced based on their characteristics. Demographic variables such as sex and age were only analysed in aggregated form to prevent identification of specific individuals, particularly given the small number of female residents.

## 3 Results

During the study period, a total of 153 262 BVC assessments were recorded out of 186 875 potential assessments (18% missing), with 7748 (5.1%) scored one or more points. Among these positive scores, 46.6% were scored as 1, 26.2% as 2 and 27.2% as 3 or higher. The most frequently observed indicators were irritability (37.6%), confusion (21.6%) and boisterousness (15.4%). These high‐risk scores (BVC ≥ 3) were most commonly recorded during the evening shift (51.1%), followed by the day shift (30.1%) and then the night shift (18.8%). In total, 1793 violent incidents were documented using the SOAS‐R, with staff being identified as the primary targets of aggression. Of these, 1325 were classified as severe incidents, defined as having SOAS‐R scores of 9 or more, constituted a smaller proportion of all violent episodes (see Table 1 for more details).

**TABLE 1:** Aggressive episodes by provocation and target (in %).

	SOAS‐R score ≥ 9 (*n* = 1325)
**Mean**	13.71 (range 9–21)
**Provocation** ^a^	
Unprovoked	34.5%
Other residents	9.4%
Helped with ADL	1.9%
Residents being denied something	22.5%
Residents being asked to do something	10.2%
Physical contact	2.7%
Unexpected situations	11.2%
I relations with change in activity	2.8%
Resident intoxicated (alcohol or illegal substances)	1.1%
Resident motivated to take prescribed medication	2.1%
Resident receives bad news	8.2%
Other reasons	15.6%
**Target of aggression** ^b^	
Nobody	0.5%
Objects	14.9%
Other residents	10.9%
Residents' self	8.5%
Staff member(s)	86.8%
Other person(s)	5.8%

Abbreviations: ADL, activities of daily living; SOAS‐R, Staff Observation Aggression Scale—Revised.

^a^ Provocations are not mutually exclusive.

^b^ Targets of aggression are not mutually exclusive.

**FIGURE 1:**
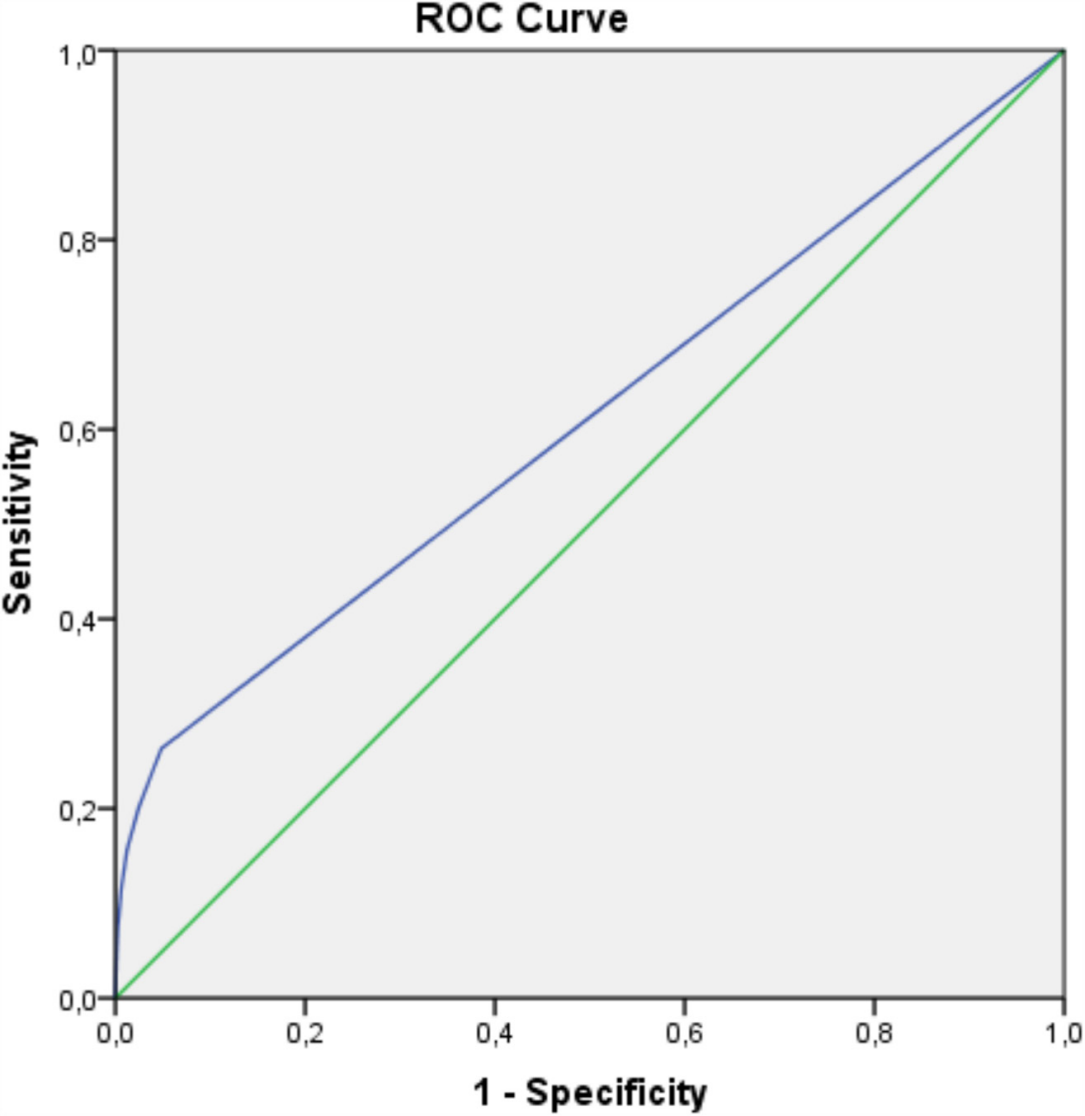
Receiver operating characteristic (ROC) curve. The ROC curve demonstrates the model's ability to predict violence (VOLDSOAS). Sensitivity (true positive rate) is plotted against 1‐specificity (false positive rate) across thresholds. The area under the curve (AUC) indicates the model's overall predictive performance.

The predictive validity of the BVC was evaluated using ROC analysis, which yielded an AUC of 0.610; this indicates a modest discriminative ability (see Figure 1). At a threshold of BVC ≥ 1, the sensitivity was 26.3%, whereas the specificity was 95.1%. This threshold resulted in a PPV of 4.9%, meaning only 4.9% of residents scoring at or above this threshold engaged in violence within the next 24 h, and a NPV of 99.3%, indicating that residents with scores below the threshold were highly unlikely to engage in violence.

At higher thresholds, such as BVC ≥ 3, sensitivity decreased to 15.7%, whereas specificity increased to 98.8%. This threshold improved PPV slightly to 10.7%, whereas NPV remained high at 99.2%. At the most restrictive threshold, BVC ≥ 6, sensitivity dropped to 4.7%, whereas specificity reached 99.9% [99.8, 99.9]. The PPV for this threshold was 24.8%, reflecting the high specificity but low overall sensitivity; the NPV remained consistent at 99.1% (see Table 2 for more details).

**TABLE 2:** Sensitivity, specificity, PPV and NPV at the different cut‐off scores for the BVC—overall.

Overall	Sensitivity	Specificity	PPV	NPV
BVC ≥ 1	0.263 [0.241, 0.285]	0.951 [0.950, 0.953]	0.049 [0.045, 0.053]	0.993 [0.992, 0.993]
BVC ≥ 2	0.203 [0.181, 0.225]	0.974 [0.973, 0.975]	0.071 [0.063, 0.075]	0.992 [0.991, 0.993]
BVC ≥ 3	0.157 [0.138, 0.176]	0.988 [0.987, 0.989]	0.107 [0.093, 0.121]	0.992 [0.991, 0.993]
BVC ≥ 4	0.116 [0.098, 0.134]	0.994 [0.993, 0.995]	0.149 [0.127, 0.171]	0.992 [0.991, 0.993]
BVC ≥ 5	0.078 [0.064, 0.092]	0.997 [0.997, 0.998]	0.208 [0.172, 0.244]	0.991 [0.990, 0.992]
BVC ≥ 6	0.047 [0.035, 0.059]	0.999 [0.998, 0.999]	0.248 [0.195, 0.301]	0.991 [0.990, 0.992]

*Note:* Numbers in [] is 95% confidence interval.

The BVC's performance varied significantly across shifts (see Table 3). During the day shift, its sensitivity ranged from 26.3% at BVC ≥ 1 to 4.2% at BVC ≥ 6, whereas its specificity ranged from 94.8% to 99.9%. During the evening shift, its sensitivity was higher, reaching 42.2% at BVC ≥ 1 and decreasing to 20.9% at BVC ≥ 6, whereas its specificity ranged from 93.3% to 98.2%. The night shift demonstrated the weakest performance, with the sensitivity dropping to 13.5% at BVC ≥ 1 and 2.4% at BVC ≥ 6, although the specificity remained high, ranging from 97.6% to 99.8%.

**TABLE 3:** Sensitivity, specificity, PPV and NPV at the different cut‐off scores for the BVC—for day, evening and night shift.

Day	Sensitivity	Specificity	PPV	NPV
BVC ≥ 1	0.263 [0.228, 0.298]	0.948 [0.943, 0.953]	0.064 [0.054, 0.074]	0.989 [0.987, 0.991]
BVC ≥ 2	0.202 [0.171, 0.233]	0.975 [0.971, 0.979]	0.098 [0.081, 0.115]	0.989 [0.986, 0.992]
BVC ≥ 3	0.146 [0.119, 0.173]	0.989 [0.986, 0.992]	0.149 [0.121, 0.177]	0.988 [0.985, 0.991]
BVC ≥ 4	0.103 [0.079, 0.127]	0.995 [0.993, 0.997]	0.217 [0.171, 0.263]	0.988 [0.986, 0.990]
BVC ≥ 5	0.067 [0.047, 0.087]	0.998 [0.997, 0.999]	0.313 [0.240, 0.386]	0.987 [0.984, 0.990]
BVC ≥ 6	0.042 [0.026, 0.058]	0.999 [0.998, 1.000]	0.384 [0.278, 0.490]	0.987 [0.984, 0.990]

Evening	Sensitivity	Specificity	PPV	NPV
BVC ≥ 1	0.422 [0.368, 0.476]	0.933 [0.928, 0.938]	0.038 [0.032, 0.044]	0.996 [0.995, 0.997]
BVC ≥ 2	0.343 [0.291, 0.395]	0.964 [0.960, 0.968]	0.057 [0.047, 0.067]	0.996 [0.994, 0.998]
BVC ≥ 3	0.285 [0.240, 0.330]	0.982 [0.978, 0.986]	0.092 [0.074, 0.110]	0.995 [0.993, 0.997]
BVC ≥ 4	0.215 [0.175, 0.255]	0.991 [0.986, 0.993]	0.128 [0.100, 0.156]	0.995 [0.993, 0.997]
BVC ≥ 5	0.142 [0.104, 0.180]	0.996 [0.994, 0.998]	0.174 [0.132, 0.216]	0.995 [0.993, 0.997]
BVC ≥ 6	0.084 [0.054, 0.114]	0.998 [0.996, 1.000]	0.209 [0.140, 0.278]	0.994 [0.992, 0.996]

Night	Sensitivity	Specificity	PPV	NPV
BVC ≥ 1	0.135 [0.103, 0.167]	0.976 [0.972, 0.980]	0.047 [0.034, 0.060]	0.992 [0.990, 0.994]
BVC ≥ 2	0.092 [0.064, 0.120]	0.987 [0.983, 0.991]	0.057 [0.040, 0.074]	0.992 [0.989, 0.995]
BVC ≥ 3	0.071 [0.047, 0.095]	0.993 [0.990, 0.996]	0.079 [0.053, 0.105]	0.993 [0.991, 0.995]
BVC ≥ 4	0.057 [0.035, 0.079]	0.996 [0.994, 0.998]	0.107 [0.065, 0.149]	0.992 [0.989, 0.995]
BVC ≥ 5	0.043 [0.025, 0.061]	0.998 [0.997, 0.999]	0.159 [0.091, 0.227]	0.992 [0.989, 0.995]
BVC ≥ 6	0.024 [0.010, 0.036]	0.999 [0.998, 1.000]	0.172 [0.086, 0.258]	0.992 [0.989, 0.995]

*Note:* Numbers in [] is 95% confidence interval.

The GLMM analysis confirmed that the BVC score was a significant predictor of violent behaviour. Each one‐point increase in the BVC score was associated with a 61% increase in the odds of violence (estimate = 0.478; odds ratio = 1.61; *p* < 0.001). The relationship between BVC score and violence risk was moderated by both sex and time of day. The interaction between BVC score and sex showed that the predictive strength of the BVC was weaker for women compared with men (estimate = −0.112; *p* = 0.003), whereas the interaction between BVC score and time of day revealed a stronger predictive power during the evening shift (estimate = 0.077; *p* = 0.055) and significantly reduced predictive strength during the night shift (estimate = −0.175; *p* < 0.001).

The model included a random intercept for each resident to account for individual variability, with a variance of 2.651 (SD = 1.628), reflecting substantial differences in the baseline violence risk among residents. Model performance metrics indicated a good overall fit, with an AIC of 13204.5 and scaled residuals ranging from −1.151 to 41.023. However, the modest AUC of 0.610 underscores the limitations in the BVC's ability to reliably predict violent behaviour.

The predicted probabilities of violence based on GLMMs varied according to sex and shift. For male residents, during the evening shift, the probability of violence increased from 1.2% at BVC = 0 to 18.2% at BVC = 6. In contrast, for female residents during the day shift, the probability of violence ranged from 0.8% at BVC = 0 to 12.7% at BVC = 6. These probabilities illustrate the influence of both sex and time of day on the BVC's predictive performance.

## 4 Discussion

This study evaluated the predictive validity of the BVC in a population of offenders with IDs residing in a specialized Danish institution. The findings demonstrate that although the BVC is a reliable tool for identifying low‐risk situations, its predictive accuracy for high‐risk situations is limited, particularly at higher thresholds and during the night shift. This raises important questions about the adaptability of the BVC to populations with IDs, where aggression and violence may manifest differently from those in psychiatric or forensic settings (Bowring et al. 2017; Gardner 2007).

The GLMM analysis confirmed that BVC scores were significantly associated with the likelihood of violent behaviour. Each one‐point increase in BVC score was linked to a 61% increase in the odds of violence, underscoring the tool's potential for assessing escalating risk. However, this predictive relationship was moderated according to sex and time of day, emphasizing the importance of contextual factors in interpreting BVC scores and tailoring intervention strategies. Given that the sample included only 10 females, and the observed differences were not large, results regarding sex differences should be interpreted with caution.

The AUC value of 0.610 in this study contrasts with the higher predictive validity reported in acute psychiatric settings, where AUC values often exceed 0.7 (Abderhalden et al. 2004; Almvik et al. 2000; Langsrud et al. 2019; Lockertsen et al. 2021; Yuniati et al. 2020). Studies in psychiatric populations have highlighted the BVC's strengths in predicting aggression but have also identified contextual factors that may limit its generalizability to ID populations. For instance, Almvik and Woods (1998) and Woods and Almvik (2002) emphasized the importance of observing short‐term behavioural cues. However, these cues may differ significantly in individuals with IDs, where aggression stems from sensory overload, communication barriers or baseline irritability (Cashin et al. 2024; Noel 2018) rather than traditional psychiatric predictors like substance use or interpersonal conflict (Weltens et al. 2021).

### 4.1 Challenges in Predictive Validity

The findings of this study align with systematic reviews, such as those by Singh et al. (2011) and Ramesh et al. (2018), which have highlighted variability in the predictive validity of risk assessment tools across different settings and populations. For example, Singh et al. (2011) reported that tools like the HCR‐20 generally achieve higher predictive validity than the BVC in forensic psychiatric populations. These differences underscore the need to adapt the BVC to better address the unique behavioural and contextual characteristics of individuals with IDs.

Moreover, the low PPV observed at higher BVC thresholds, especially during the night shift, reflects broader challenges in applying structured risk assessment tools in environments with low base rates of violence (Ramesh et al. 2018). In such settings, the consequences of false positives,such as unnecessary interventions,must be carefully weighed against the risk of missing violent incidents.

### 4.2 Temporal and Environmental Variability in BVC Performance

The findings reveal substantial variability in the BVC's predictive performance across shifts and between sexes. Evening shifts demonstrated the highest sensitivity and specificity, which aligns with the findings of Lockertsen et al. (2020) and van de Sande et al. (2011); this likely because of increased observational opportunities. Meanwhile, night shifts highlighted limitations inherent in low‐activity periods. These studies emphasize the fact that periods of structured activity and increased staff–resident interactions allow for better observation of escalating behaviours, leading to improved predictive accuracy. For example, van de Sande et al. (2011) demonstrated that integrating short‐term risk assessments into daily care routines can reduce aggression incidents and seclusion durations.

In contrast, the poor performance of the BVC during night shifts reflects well‐documented challenges in low‐activity environments (Lockertsen et al. 2020; Maguire et al. 2018). Reduced staffing levels and fewer observational opportunities contribute to the lower sensitivity, as is also observed in this study. Evidence suggests that supplementing the BVC with complementary tools, such as the Kennedy Axis V or patient self‐reports, could enhance predictive performance during periods of low activity (Lockertsen et al. 2020; van de Sande et al. 2011).

### 4.3 Sex‐Specific Differences

The weaker predictive relationship for female residents highlights a critical limitation of the BVC in identifying nonphysical forms of aggression, such as verbal outbursts or self‐harm. Similar findings have been reported in studies, such as that from Eriksen et al. (2018), which advocate for the integration of supplemental indicators tailored to women. This is particularly relevant in mixed‐gender settings, where aggression patterns differ significantly between men and women (Archer et al. 2016; Morton et al. 2022).

### 4.4 Strengths and Limitations of the Study

This study offers several key strengths that enhance the reliability and relevance of its findings. By focusing on a unique population of offenders with IDs, it addresses an important gap in violence risk assessment research, exploring the predictive validity of the BVC in a nonpsychiatric setting. The use of a large dataset comprising over 153 000 BVC assessments and 1325 violent incidents ensures robust statistical power and allows for detailed subgroup analyses according to shift and sex, providing valuable insights into contextual and temporal variability.

Methodologically, the use of GLMMs is a notable strength, as it accounts for the repeated measures design and individual variability in violence risk. This approach enabled the analysis of interactions between BVC scores, sex and time of day, adding depth to the findings. Additionally, the use of real‐world data, which were collected as part of routine care, enhances the external validity of the results, making them directly applicable to similar institutional contexts.

However, this study also has its limitations. Conducting the research within a single institution limits the generalizability of the findings to other settings, such as psychiatric hospitals or correctional facilities. The reliance on staff‐reported SOAS‐R data introduces potential reporting bias, particularly during night shifts, where reduced staffing levels are observed. Moreover, the BVC, although validated for psychiatric populations, may not fully capture the unique behavioural characteristics of individuals with IDs, such as sensory sensitivities or communication challenges, which could impact its predictive accuracy.

The observational nature of the study precludes causal inferences, and unmeasured variables, such as environmental conditions or staff–resident dynamics, may have influenced the results. Additionally, multicollinearity among the BVC's six behavioural indicators (*confused*, *irritable*, *boisterous*, *verbal threats*, *physical threats* and *attacking objects*) posed challenges in isolating the effects of individual predictors, potentially reducing model precision.

Despite these limitations, this study provides valuable insights into the utility and constraints of the BVC in a specialized population. It highlights the need for adapting risk assessment tools to better address the unique characteristics of individuals with IDs, contributing to the development of more tailored and effective violence prevention strategies.

## 5 Implications for Practice

This study highlights several important implications for the practical use of the BVC in institutions for individuals with IDs. The evening shift, with its higher sensitivity and PPV, presents the greatest opportunity for proactive prevention of violence. Targeted training for staff during this period could emphasize the early recognition of high‐risk behaviours. In contrast, given the BVC's limited performance during the night shift, alternative approaches, such as real‐time monitoring technologies or increased staffing, may be necessary to improve risk prediction. The observed gender differences highlight the need for sex‐specific adaptations to the BVC or the integration of additional behavioural markers tailored to women (Eriksen et al. 2018). Future research should further explore sex‐based differences in aggression within larger and more balanced samples to strengthen the evidence base for gender‐sensitive modifications to the BVC. Additionally, future efforts should focus on developing and validating risk assessment tools that incorporate factors that are more relevant to the population in question (Ramesh et al. 2018; Singh et al. 2011), such as sensory sensitivities, communication difficulties and baseline irritability.

### 5.1 Future Research Directions

Future research should aim to validate these findings across multiple institutions and explore the development of population‐specific risk assessment tools. Additionally, studies integrating real‐time monitoring technologies or combining the BVC with other validated tools may enhance the predictive accuracy across shifts (Lockertsen et al. 2020; van de Sande et al. 2011). Research should also consider the role of environmental factors, such as staffing ratios and activity levels, in shaping tool performance.

## 6 Conclusions

This study highlights the strengths and limitations of the BVC when applied to individuals with IDs in a specialized institutional setting. Although the tool is reliable for ruling out violence, its limitations in predicting high‐risk situations underscore the need for tailored adaptations and complementary assessment strategies. These findings contribute to a growing body of literature that emphasizes the importance of contextual and population‐specific approaches to violence risk assessments. Future efforts should focus on addressing these challenges to improve safety and care for residents and staff alike.

## Ethics Statement

This study was conducted in accordance with ethical guidelines and standards for research involving human participants. The study was reviewed and approved by the Danish Data Protection Agency. Written informed consent was not obtained from the individual(s) for the publication of any potentially identifiable images or data included in this article. Measures were taken to ensure confidentiality, and only authorized personnel had access to sensitive information. The research adhered to the principles of beneficence, respect for persons, and justice as outlined in the Declaration of Helsinki.

## Conflicts of Interest

The authors declare no conflicts of interest.

## Data Availability

The data that support the findings of this study are available on request from the corresponding author. The data are not publicly available because of privacy or ethical restrictions.
